# Application-Aware Anomaly Detection of Sensor Measurements in Cyber-Physical Systems

**DOI:** 10.3390/s18082448

**Published:** 2018-07-27

**Authors:** Amin Ghafouri, Aron Laszka, Xenofon Koutsoukos

**Affiliations:** 1 Department of Electrical Engineering and Computer Science, Institute for Software Integrated Systems (ISIS), Vanderbilt University, Nashville, TN 37212, USA ; aminghafouri.ut@gmail.com; 2 Department of Computer Science, University of Houston, Houston, TX 77204, USA; alaszka@uh.edu

**Keywords:** anomaly detection, detection error, cyber-physical systems, traffic sensors

## Abstract

Detection errors such as false alarms and undetected faults are inevitable in any practical anomaly detection system. These errors can create potentially significant problems in the underlying application. In particular, false alarms can result in performing unnecessary recovery actions while missed detections can result in failing to perform recovery which can lead to severe consequences. In this paper, we present an approach for *application-aware anomaly detection (AAAD)*. Our approach takes an existing anomaly detector and configures it to minimize the impact of detection errors. The configuration of the detectors is chosen so that application performance in the presence of detection errors is as close as possible to the performance that could have been obtained if there were no detection errors. We evaluate our result using a case study of real-time control of traffic signals, and show that the approach outperforms significantly several baseline detectors.

## 1. Introduction

Sensors deployed in cyber-physical systems (CPS) for monitoring and control purposes are prone to anomalies (e.g., reliability failures and cyber-attacks). To detect anomalies and prevent their harmful effects, anomaly detection systems (ADS) are utilized. However, ADS suffer from false positives (i.e., false alarms) and false negatives (i.e., missed detections), which may result in high performance degradation in CPS applications. In particular, false positives result in recovery that is not required, and false negatives result in failing to perform recovery when it is indeed required. Such detection errors can cause incorrect measurements being transmitted to a controller, and thus result in obtaining non-optimal or even destabilizing control decisions, which may compromise the performance of the system. For example, detection errors may result in disastrous events, such as reactor explosion in process control systems, water contamination in water distribution networks, and extremely heavy traffic congestion in intelligent transportation systems [1,2].

To address the challenges caused by detection errors, it is necessary to take into account the CPS application when designing anomaly detectors, and to quantify the losses in the application caused by potential detection errors. To minimize the losses, it is desirable to reduce the detection errors as much as possible. However, there exists a trade-off between them (i.e., decreasing the rate of false alarms may increase the rate of missed faults, and vice versa), which can be changed through a detection threshold. Therefore, the performance loss caused by detection errors can be minimized by selecting the right detection threshold.

Our goal is to perform these steps using a novel approach, which takes an existing anomaly detector and configures it considering the behavior of the controller. We call our framework *Application-Aware Anomaly Detection (AAAD)*. This framework takes into account the interactions between the controller and the application, and so it can compute how each detection decision may affect the underlying application. Knowing this, the detector attempts to make detection decisions that will result in the least performance loss in the underlying application if the detection decision is not accurate due to false positive and false negative errors.

Previous works have proposed different anomaly detection methods for CPS [1]. In addition, there is a wide body of literature on machine learning-based anomaly detection [3]. The problem of finding optimal thresholds for intrusion detectors is studied in [4]. The paper shows that computing optimal attacks and defenses is computationally expensive, and proposes heuristic algorithms for computing near-optimal strategies. Further, the work in [5] studies the problem of finding optimal thresholds for anomaly-based detectors implemented in dynamical systems in the face of strategic attacks. The paper provides algorithms to compute optimal thresholds that minimize losses considering best-response attacks. However, there is little work that takes into account the tight interaction between the detector and the controller of a CPS, which as we show in this work if taken into account, can result in improved performance and robustness.

Contributions

In this paper, we propose an application-aware anomaly detection framework for minimizing the impact of detection errors in CPS. The contributions of AAAD are as follows:

1. We devise an effective detector for identifying anomalies in sensor measurements using machine learning regression and an approach to recover from anomalies in order to maintain operation when detection alerts are triggered.
2. We formulate the AAAD problem, in which a detector is optimally configured such that the performance loss in the presence of detection errors is minimized. In particular, the thresholds are selected so that the performance of the system in the presence of detection errors is as close as possible to the performance that could have been achieved if there were no detection errors.
3. We show that the AAAD problem is computationally challenging, and then we present an efficient algorithm to find near-optimal solutions.
4. We analyze two special cases of the application-aware detection problem: (a) single detector and (b) detectors with equal thresholds. We present optimal solutions for both special cases which can provide insights into the novelty of the approach.
5. We evaluate AAAD using simulation experiments on a case study of real-time control of traffic signals. The evaluation results demonstrate the benefits of the approach compared with standard anomaly detection techniques.

We believe that the proposed approach can be useful in any system where there are a significant number of sensors with high variations in sensor values, which may cause many false-positive and false-negative errors. A real-world example of such a CPS application would be real-time control of traffic signals since in large cities, there are thousands of sensors that could become anomalous. Throughout the paper, we will use traffic sensors as a running example to illustrate the application of our framework, but we present the model and results using a general, domain-agnostic formulation. Alternative examples of possible application domains include sensors in water-distribution networks [6].

Related Work

Anomaly detection in cyber-physical systems presents an important and challenging problem [7,8]. As a result, a variety of approaches have been proposed in prior work on anomaly detection. In contrast to our approach, these prior results focused on the design of detectors rather than the optimal configuration of an existing detector.

Several detectors have been built on machine learning and, in particular, neural networks. Goh et al. present an unsupervised approach for anomaly detection in cyber-physical systems based on recurrent neural networks and the cumulative sum method [9]. Kosek presents a contextual anomaly detection method for smart grids based on neural networks [10]. Krishnamurthy et al. use an alternative model, Bayesian networks [11]. They present an approach for learning causal relations and temporal correlations in cyber and physical variables from unlabeled data using Bayesian networks. These networks can then be used to detect anomalies and isolate their root causes.

Jones et al. propose a formal-methods-based approach for anomaly detection in cyber-physical systems [12]. They introduce a model-free, unsupervised learning procedure that constructs a signal temporal logic (STL) formula from system output data collected during normal operations. Then, anomalies can be detected by flagging system trajectories that do not satisfy the learned formula. In a follow-up work, Kong et al. describe a formal-methods-based approach for supervised anomaly learning [13]. Chibani et al. investigate the problem of designing fault-detection filters for fuzzy systems, considering faults and unknown disturbances in discrete-time polynomial fuzzy systems [14,15]. A diagnostic observer-based system for fault detection of fuzzy systems which optimizes the worst case robustness and fault sensitivity is presented in [16].

Anomaly detection has also been considered in the context of security intrusions, that is, to detect cyber-physical attack against a CPS [17]. For example, Urbina et al. study physics-based detection of stealthy attacks against industrial control systems [1]. They review prior work on attack detection and argue that many of these use detection schemes that do not limit the impact of stealthy attacks. They then propose a new metric for measuring impact and demonstrate that attacks may be detected with a proper configuration. In contrast, Kleinmann and Wool consider detection of attacks against industrial control systems based on cyber anomalies [18].

Besides anomaly detection, other approaches have also been considered for detecting faults or attacks in traffic networks. Lu et al. review prior work on the problem of anomaly detection of traffic sensors [19]. Based on the level of data used, they divide detection methods into three levels: macroscopic (highly-aggregated data), mesoscopic (synchronized data for a section of freeways), and microscopic. They also review data-correction methods and provide practical guidelines for anomaly detection in traffic applications. Zygouras et al. present three methods—based on Pearson’s correlation, cross-correlation, and multivariate ARIMA—to detect faulty traffic measurements [20]. They discuss the performance of all three methods and demonstrate that they are complementary to each other. Further, they employ crowd-sourcing to resolve whether irregular measurements are due to faulty sensors or unusual traffic. Finally, Robinson presents a test, based on the relationship between flows at adjacent sensors, to detect faulty loop detectors [21].

Nonetheless, to the best of our knowledge, none of these papers consider the performance of the controller in the design and configuration of anomaly detectors, and they do not perform any application-aware optimization to improve detection performance.

Paper Outline

The rest of this paper is organized as follows. In Section 2, we introduce the system model. In Section 3, we discuss the regression-based anomaly detection. In Section 4, we present the application-aware detection problem for detection error-tolerant selection of thresholds in anomaly detectors. In Section 5, we analyze the application-aware detection problem and present an algorithm to obtain near-optimal solutions. In Section 6, we study two special variations of the application-aware detection problem, that is, single detector and detectors with equal threshold. In Section 7, we evaluate our approach numerically using a case study of real-time control of traffic signals. Finally, we offer concluding remarks in Section 8.

## 2. Model

In this section, we present the system model and informally introduce the problem of application aware anomaly detection. We also present a running example of real-time control of traffic signals that is used throughout the paper to demonstrate our approach.

### 2.1. Notation

Vectors are denoted by bold symbols. Vector y at timestep *k* is described by $y^{k}$. We omit the timestep symbol when all symbols have same timestep *k*. However, timestep symbol is used when there are different timesteps present or when it eases understanding. Given vector y and set of indices *I*, vector $y_{I}$ is defined as a vector with same size as y that has the same size as y for indices in *I*, and is zero otherwise. For a list of symbols used in this section, see Table 1.

### 2.2. System Model

**Figure 1:**
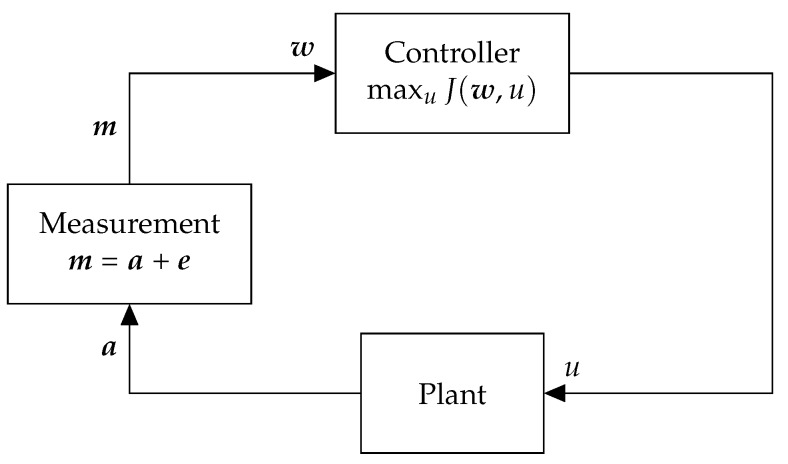
System Model. Please note that in this case w=m.

Consider a CPS, for example, an intelligent transportation system or a process control system, consisting of a plant and a controller as shown in Figure 1. At each timestep, given measurements w containing information about the system, a controller computes a control input *u* that maximizes a utility function J(w,u). In other words, the controller determines the optimal control input $u^{*}$ defined as

(1) $$u^{*}\in \underset{u}{\mathrm{argmax}}J\left(w,u\right)\;,$$

where the optimal utility is denoted by $J^{*}\left(w\right)$.

#### Anomalous Sensors

Sensors may be anomalous due to hardware failures or sensor attacks. If sensor s∈S is anomalous, there is a discrepancy between the actual and observed measured values. In other words, if $a_{s}$ is the actual value and $m_{s}$ is the observed measurement at a timestep, for an anomalous sensor we have $m_{s}=a_{s}+e_{s}$, where $e_{s}\in R$ is the error value at that timestep.

### 2.3. Example: Real-Time Traffic Signal Control

We present real-time control of traffic signals as a running example that is used throughout the paper to demonstrate the applicability of our approach. In the following, we describe the widely-popular max-pressure controller for optimal control of traffic signals [22]. In the original max-pressure algorithm presented in [22], the traffic state is represented using exogenous demands that are then routed through the network using routing ratios. In this work, instead of using exogenous demands that are then transformed to internal demands through using routing ratios, we assume that the internal demands are directly provided. Please note that this does not affect the max-pressure algorithm as the algorithm effectively uses internal demands in its computations.

#### Max-Pressure Controller

Consider a network of intersections *I* with road links *L*. Movement from a link i∈L to a link j∈L is denoted by a pair (i,j)∈E. Further, let each movement (i,j) have a queue associated with it, and at each timestep, let x(i,j) represent the length of this queue. The length of the queue shows how many vehicles intend to travel from *i* to *j*. For each movement (i,j), the pressure is defined as

$$P\left(i,j\right)=x\left(i,j\right)-\sum_{p}x\left(j,p\right)\;,$$

which is simply the number of cars in the queue minus the total number of cars in the downstream queues.

Each intersection *n* has a traffic signal with a set of admissible stages $\Phi_{n}$. Each stage $u_{n}\in \Phi_{n}$ is a set of simultaneous movements that are permitted by the traffic signal. If $u_{n}$ permits a movement (i,j), then $u_{n}\left(i,j\right)=1$, otherwise $u_{n}\left(i,j\right)=0$. Let c(i,j) be the saturation flow of movement (i,j). Given a stage $u_{n}\in \Phi_{n}$, pressure-release (i.e., utility) for intersection *n* is defined as

$$J_{n}\left(u_{n}\right)=\sum_{i,j}c\left(i,j\right)\;P\left(i,j\right)\;u_{n}\left(i,j\right)\;.$$

Traffic sensors can be used to measure x(i,j) for all (i,j)∈E and they are prone to failures.

Algorithm 1 presents the max-pressure (MP) controller in detail. At each intersection *n*, the MP controller selects the stage $u_{n}$ that results in the maximum pressure-release. In other words, the MP controller computes

(2) $$u_{n}^{*}\in \underset{u_{n}\in \Phi_{n}}{\mathrm{argmax}}\sum_{i,j}c\left(i,j\right)\;P\left(i,j\right)\;u_{n}\left(i,j\right)\;.$$

Please note that at each intersection, the MP control selects a stage that depends only on the queues adjacent to the intersection. It is shown that the MP controller maximizes network throughput [22].

**Algorithm 1** Max-Pressure Controller [22]. **Input:**x(i,j) for all (i,j)∈E
1:**for all**n∈I**do**2:    **for all** (i,j)∈E **do**3:        $P\left(i,j\right)\leftarrow x\left(i,j\right)-\sum_{p}x\left(j,p\right)$4:    **end for**5:    $u_{n}^{*}\leftarrow {\mathrm{argmax}}_{u_{n}\in \Phi_{n}}\sum_{i,j}c\left(i,j\right)\;P\left(i,j\right)\;u_{n}\left(i,j\right)$6:**end for**7:**return**${\left\{u_{n}^{*}\right\}}_{n\in I}$


The overall utility for traffic network can be calculated by adding individual utilities for the intersections. That is, $J\left(x,u\right)=\sum_{n}J_{n}\left(x,u_{n}\right)$ where $u={\left\{u_{n}\right\}}_{n\in I}$. Please note that using this representation, the MP optimization problem becomes the same as (1).

### 2.4. Anomaly Detection, Recovery, and Resilience

Next, we discuss anomalies caused by sensor faults and their detection, and we informally introduce the problem of optimal detector configuration, which we will formalize in Section 4.4. In contrast to the previous subsection, where we introduced our running example, the discussion here will again be domain agnostic.

Anomalies may cause damage to the system and significantly degrade the performance of the CPS application (as measured by the utility function J(w,u)). Consequently, we must employ an anomaly detector to detect faults in sensor measurements. In our running example, anomalies can affect the traffic measurements, and therefore, degrade the utility of the traffic signal control. Suppose that we have an anomaly detection method. Upon detection, the system must recover from anomalies and continue operation. To this end, we must employ a recovery method in order to continue operation in the presence of detection alerts. In the traffic signal control example, operation must continue even in the presence of anomalous traffic measurements.

Now, suppose that we have a recovery approach that computes a recovered vector of measurements in the presence of detection alerts. In anomaly detectors, there are detection errors, that is, false positives (i.e., false alarms) and false negatives (i.e., missed faults). If there were no detection errors, the recovered vector of measurements would be close to the actual values (of course assuming that the recovery approach works well). However, in the presence of detection errors, false positives result in recovery that is not required, and false negatives result in failing to perform recovery when it is indeed needed.

To illustrate the effect of detection errors on the application, let $w^{\prime }$ denote the recovered measurement vector, which will result in the utility $J^{\prime }=\max_{u}J\left(w^{\prime },u\right)$. However, if there were no detection error, we could have obtained the optimal utility $J^{*}=\max_{u}\left(a,u\right)$. Hence, we face the problem of optimally configuring an anomaly detector through the selection of detection thresholds so that the actual obtained utility in the presence of detection errors (i.e., $J^{\prime }$) is as close as possible to the utility that would have been obtained if there were no detection errors (i.e., $J^{*}$). In the traffic signal control example, the utility is quantified by the pressure release for an intersections that can be used as a proxy for maximizing network throughput.

## 3. Anomaly Detection

In this section, we construct an example regression-based anomaly detector for identifying anomalous sensor measurements. We then discuss detection errors and some metrics that are used to characterize them.

### 3.1. Regression-Based Anomaly Detector

To protect the system against anomalies, we must detect them quickly and accurately. Many different anomaly detection systems have been proposed in the literature. For a comprehensive review of anomaly detection methods, we refer the reader to [3] for machine-learning-based detectors and [1] for detectors used in CPS. In this work, we use regression-based anomaly detectors because in addition to high detection performance, such detectors require no knowledge of the physical system, can take into account complex and nonlinear behaviors of the system, and are easy to implement and can be highly scalable.

#### 3.1.1. Architecture

**Figure 2:**
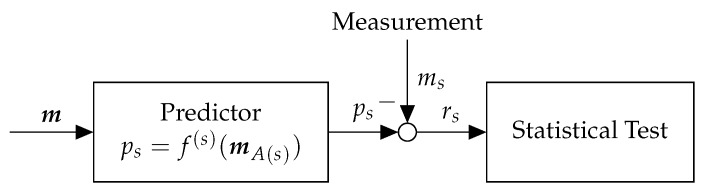
Regression-Based Anomaly Detector.

Figure 2 shows the architecture of regression-based anomaly detector. The detector consists of two main components: (1) Predictor and (2) Statistical Test. The predictor predicts the value of a sensor given some information about the system state (e.g., current value of other sensors, previous control inputs). Then, the statistical test compares the computed prediction to the observed measurement and decides whether the sensor is normal or anomalous. We describe each component in more detail considering the running example of real-time traffic control.

#### 3.1.2. Predictor

Our goal is to find a function $f^{\left(s\right)}$ that maps spatial or temporal features to the actual value of a sensor *s* (e.g., traffic flow or occupancy). In practice, two traffic sensors are highly correlated if they are in close proximity. Thus, we let the features be the measured values of other adjacent sensors at the same timestep, denoted by $m_{A(s)}$ where A(s) is a set of sensors adjacent to A(s) found using cross-validation. Please note that this approach is particularly applicable to traffic networks as there are usually many redundant sensors in the network. The function $f^{\left(s\right)}$ can then be obtained using suitable machine learning regression algorithm such as deep neural networks [23], Gaussian Processes [24], and many others [25]. Thus, for sensor *s*, we obtain the prediction as $p_{s}=f^{\left(s\right)}\left(m_{A(s)}\right)$.

#### 3.1.3. Statistical Test

The statistical test efficiently detects anomalies for each sensor s∈S by comparing the measured value $m_{s}\left(k\right)$ with the predicted value $p_{s}\left(k\right)$. Given a set of measured values $m={\langle m_{s}\rangle }_{s\in S}$ and predicted values $p=\;{\langle p_{s}\rangle }_{s\in S}$, residual signals are computed as r=|m−p|. Then, given the residuals, the statistical test makes detection decisions $d={\langle d_{s}\rangle }_{s\in S}$, where for each sensor *s*, the decision $d_{s}$ is either normal or anomalous.

Different detection algorithms can be used to implement the statistical test [26]. In this work, we consider a stateless threshold-based detector defined as follows. It should be noted that for ease of presentation, our model assumes that a detector can be configured using a single threshold value $\tau_{s}$. However, our results can be easily applied to detectors that are configured using multiple parameter values. We discuss how to incorporate such detectors into our framework in Section 3.2. Given detection thresholds $\tau ={\langle \tau_{s}\rangle }_{s\in S}$, for each sensor *s*, if the residual $r_{s}$ is less than or equal to the threshold $\tau_{s}$, then *s* is marked normal and otherwise, *s* is marked anomalous. Thus

(3) $$\begin{array}{c} d_{s}=\left\{\begin{array}{cc} \mathrm{normal}\;(s\in N) & \;\mathrm{if}\;r_{s}\leq \tau_{s}\\ \mathrm{anomalous}\;(s\in A) & \;\mathrm{otherwise} \end{array}\right.. \end{array}$$

### 3.2. Detection Error

In anomaly detectors, there might be a *false negative*, which means failing to raise an alarm when an anomaly did happen. Further, there might be a *false positive*, which means raising an alarm when the system exhibits normal behavior. It is desirable to reduce the false positive and false negative probabilities as much as possible. However, there exists a trade-off between them, which can be controlled by changing the detection threshold. In particular, by decreasing (increasing) the threshold, one can decrease (increase) the FN probability and increase (decrease) the FP probability.

We represent the FN probability for each sensor *s* by the function $F\;N_{s}:\;R_{+}\;\to \;\left[0,1\right]$, where $F\;N_{s}\left(\tau_{s}\right)$ is the probability of FN when the threshold is $\tau_{s}$, given that the sensor is anomalous. Similarly, we denote the attainable FP probability for each sensor *s* by $F\;P_{s}:\;R_{+}\to \;\left[0,1\right]$, where $F\;P_{s}\left(\tau_{s}\right)$ is the FP probability when the threshold is $\tau_{s}$, given that the sensor is in normal operation. The true positive and true negative probabilities are also denoted by $T\;P_{s}\left(\tau_{s}\right)$ and $T\;N_{s}\left(\tau_{s}\right)$. Clearly, we have $T\;P_{s}\left(\tau_{s}\right)=1-F\;N_{s}\left(\tau_{s}\right)$ and $T\;N_{s}\left(\tau_{s}\right)=1-F\;P_{s}\left(\tau_{s}\right)$.

It should be noted that even though we assumed in Section 3.1.3 that each detector can be configured using a single threshold value $\tau_{s}$, our framework can actually be applied to detectors that are configured using multiple parameter values. For such a detector, each possible configuration (i.e., combination of parameter values) results in some pair of false-negative and false-positive error probabilities. By considering the Pareto optimal configurations (i.e., configurations such that neither probability can be decreased without increasing the other), we can obtain a curve that represents the best attainable pairs of false-negative and false-positive probabilities. Then, we can simply let threshold values correspond to points on this curve such that false-negative and false-positive probabilities ($F\;N_{s}$ and $F\;P_{s}$) are increasing and decreasing functions of the threshold $\tau_{s}$, respectively.

## 4. Application-Aware Anomaly Detection

In this section, we present the problem of application-aware anomaly detection (AAAD). First, we describe an approach for recovery in order to continue operation in the presence of detection alerts. Then, we quantify the utility losses in the application caused by potential detection errors. Based on the characterization of the utility losses, we formulate the AAAD problem, i.e., the problem of finding detection thresholds so that the obtained utility in the presence of detection errors is as close as possible to the utility that could have been obtained if there were no detection errors.

### 4.1. Architecture

**Figure 3:**
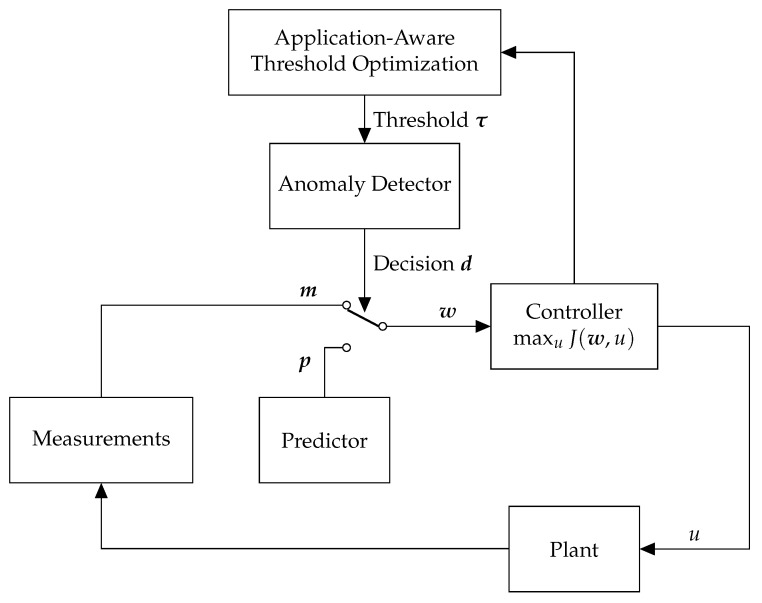
Architecture of Application-Aware Anomaly Detection.

Figure 3 shows the AAAD architecture. If there is a detection alert, the prediction is routed to the application, instead of the measurement. The threshold of each detector is selected such that in the presence of detection error, the routed value (i.e., measurement or prediction) still obtains a utility close to the utility that could have been obtained if there were no detectors. (Please note that in the figure, the predictor is not connected to the anomaly detector since this framework is applicable to any threshold-based detector, and not only regression-based detectors.)

### 4.2. Recovery

We consider a recovery approach in order to continue operation in the presence of detection alerts. If sensor *s* is marked normal, then the observed measurement $m_{s}$ is transmitted to the controller. However, if sensor *s* is marked anomalous, then the observed measurement is discarded and instead, the prediction $p_{s}$ is transmitted to the controller. The switch in Figure 3 illustrates this idea. To formally represent the recovery approach, let $w_{s}$ denote the *recovered measurement* transmitted to the controller. Then, $w_{s}$ can be described as

(4) $$\begin{array}{c} w_{s}=\left\{\begin{array}{cc} m_{s} & \;\mathrm{if}\;s\;\mathrm{is}\;\mathrm{normal}\\ p_{s} & \;\mathrm{if}\;s\;\mathrm{is}\;\mathrm{anomalous} \end{array}\right.. \end{array}$$

For the threshold-based detector defined by (3), the measurement of sensor *s* is marked normal if $|p_{s}-\;m_{s}|\leq \tau_{s}$ and anomalous otherwise. Therefore, for threshold-based detectors, the above equation can be re-written as

(5) $$\begin{array}{c} w_{s}\left(\tau_{s}\right)=\left\{\begin{array}{cc} m_{s} & \;\mathrm{if}\;|m_{s}-p_{s}|\leq \tau_{s}\\ p_{s} & \;\mathrm{otherwise}\; \end{array}\right.. \end{array}$$

Please note that in this case, given prediction $p_{s}$ and measurement $m_{s}$, the value of $w_{s}$ depends on the threshold $\tau_{s}$. To highlight this dependence, we use the notation $w_{s}\left(\tau_{s}\right)$ instead of $w_{s}$. To summarize, given vectors of predictions p, measurements m, and thresholds τ, using (5), we are able to compute the recovered measurement vector w(τ) that is transmitted to the controller.

We assume that when a measurement is normal, it provides the best obtainable value for the sensor. Also, we assume that when a measurement is anomalous, the prediction provides the best obtainable value for the sensor.

### 4.3. Worst-Case Utility Loss Due to Detection Error

The control input *u* (i.e., defined by  (1)) depends on the recovered measurements w(τ) (i.e., defined by (5)), and the recovered measurements w(τ) depend on the detection thresholds τ. Therefore, the value of control input depends on thresholds τ. For example, if the thresholds are small (large), there will be many (few) detection alarms, and so predictions (measurements) will often be transmitted to the controller. Unfortunately, this will be problematic in the presence of detection errors.

Given threshold τ, let *N* be the set of sensors that are marked normal (i.e., $\forall s\in N,r_{s}\leq \tau_{s}$) and let *A* be the set of sensors that are marked anomalous (i.e., $\forall s\in A,r_{s}>\tau_{s}$). Based on the recovery method (5), the predictions are used for marked-anomalous sensors in *A* and measurements are used for marked-normal sensors in *N* to create the recovered measurement vector, i.e., $w\;=\;p_{A}\;\cup \;m_{N}$. Next, given the recovered measurements $p_{A}\cup m_{N}$, the controller computes the control input $u_{0}\;\in \;{\mathrm{argmax}}_{u}J\left(p_{A}\cup \;m_{N},u\right)$, concisely denoted by $U(p_{A}\cup m_{N})$. This is expected to obtain the utility $J(p_{A}\cup m_{N},u_{0})$. However, the expected utility is obtained only if there is no detection error. Unfortunately, if there is a detection error, a different and potentially much lower utility is obtained.

#### 4.3.1. Obtained Utility vs. Optimal Utility

We now quantify the actual obtained utility in presence of detection errors. Let fp⊆A be the set of false positives, that is, sensors in fp are normal but they are marked anomalous. Since these sensors are normal, the measurements $m_{fp}$ should have been transmitted to the controller, but due to false positives, the predictions were mistakenly transmitted. Similarly, let fn⊆N be the set of false negatives, that is, sensors in fn are anomalous but they are marked normal. Since these sensors are anomalous, the predictions $p_{fn}$ should have been transmitted to the controller but the measurements were mistakenly transmitted. Hence, for the control input $u_{0}=U\left(p_{A}\cup m_{N}\right)$ computed above, the obtained utility will actually be $J(p_{tp}\cup m_{fp}\cup m_{tn}\cup p_{fn},u_{0})$. On the other hand, if there were not detection errors, the optimal control input would have been $u^{*}\in {\mathrm{argmax}}_{u}J\left(p_{tp}\cup m_{fp}\cup m_{tn}\cup p_{fn},u\right)$, concisely denoted by $U(p_{tp}\cup m_{fp}\cup m_{tn}\cup p_{fn})$.

#### 4.3.2. Utility Loss

To put this all together, given decisions *A* and *N* (computed given r and τ as (3)), and the detection performance sets tp, fp, tn, and fn, the probability of occurrence of such detection error scenario is

(6) $$\mathrm{Pr}\left(\tau ,tp,fp,tn,fn\right)=\prod_{s\in tp}T\;P_{s}\left(\tau_{s}\right)\cdot \prod_{s\in fp}F\;P_{s}\left(\tau_{s}\right)\cdot \prod_{s\in tn}T\;N_{s}\left(\tau_{s}\right)\cdot \prod_{s\in fn}F\;N_{s}\left(\tau_{s}\right)\;.$$

As discussed above, in this case, we could have obtained the optimal utility $J(p_{tp}\cup m_{fp}\cup m_{tn}\cup p_{fn},U\left(p_{tp}\cup m_{fp}\cup m_{tn}\cup p_{fn}\right))$, but we obtained the smaller utility $J(p_{tp}\cup m_{fp}\cup m_{tn}\cup p_{fn},U\left(p_{A}\cup m_{N}\right))$. Thus, we incurred a utility loss of

(7) $$\begin{array}{c} \Delta J=\underset{\mathrm{Optimal}\;\mathrm{Utility}}{\underset{︸}{J^{*}\left(p_{tp}\cup m_{fp}\cup m_{tn}\cup p_{fn}\right)}}-\underset{\mathrm{Obtained}\;\mathrm{Utility}}{\underset{︸}{J(p_{tp}\cup m_{fp}\cup m_{tn}\cup p_{fn},U\left(p_{A}\cup m_{N}\right))}}\;. \end{array}$$

Hence, the expected utility loss of detection error scenario tp⊆A, fp=A−tp, tn⊆N, and fn=N−tn is

(8) C(τ,tp,fp,tn,fn)=Pr(τ,tp,fp,tn,fn)·ΔJ.

where Pr(τ,tp,fp,tn,fn) is obtained using (6) and ΔJ is obtained using (7).

#### 4.3.3. Worst-Case Analysis

Since the sets of false positives and false negatives are not know a priori, we need to consider any possible scenario. We define the worst-case loss due to detection errors as follows.

**Definition** **1** (Worst-Case Detection Error Loss). *Given the thresholds **τ** and the residuals r, the worst-case loss due to detection errors is defined as*

(9) $$\begin{array}{cc} L\left(\tau \right)=\max_{\begin{array}{c} tp\subseteq A,tn\subseteq N\\ fp=A-tp\\ fn=N-tn \end{array}}C(\tau ,tp,fp,tn, & fn)\;, \end{array}$$

*where C(τ,tp,fp,tn,fn) is defined as (8), and A and N are found using (3).*

### 4.4. Optimal Application-Aware Anomaly Detection Problem

To protect against the utility loss due to detection errors, the designer must choose the thresholds that result in the best performance with respect to the worst-case loss (9). An application-aware anomaly detector achieves this by finding the optimal thresholds $\tau^{*}$ in each time step. We call this problem the Application-Aware Anomaly Detection Problem:

**Definition** **2** (Application-Aware Anomaly Detection Problem). *Given a system model, an anomaly detector, and measured and predicted sensor values, the Application-Aware Anomaly Detection Problem is finding the optimal thresholds $\tau^{*}$ that minimizes the loss (9); in other words,*

(10) $$\tau^{*}\in \underset{\tau }{\mathrm{argmin}}L\left(\tau \right)\;.$$

If we are not able to change the thresholds at each timestep, and instead can change thresholds every *T* timesteps, we define

(11) $$\bar{L}\left(\tau \right)=\frac{1}{T}\sum_{k=1}^{T}L^{k}\left(\tau \right)\;,$$

and then we find thresholds $\tau^{*}$ that minimize the above equation. We call this problem the Static Application-Aware Anomaly Detection Problem:

**Definition** **3** (Static Application-Aware Anomaly Detection Problem). *Given a system model, an anomaly detector, and measured and predicted sensor values, the Static Application-Aware Anomaly Detection Problem in a time period T is finding the optimal thresholds $\tau^{*}$ that minimizes the loss (11):*

(12) $$\tau^{*}\in \underset{\tau }{\mathrm{argmin}}\bar{L}\left(\tau \right)\;.$$

Clearly, (10) can be solved as a special case of (12) for T=1.

## 5. Analysis

In this section, we solve the problems (10) and (12). First, we analyze the problem of worst-case detection error loss (9), and we prove that solving this problem is computationally challenging. We then present Algorithm 2 which is an efficient algorithm to obtain approximately optimal solutions. Second, we present Algorithm 3 to solve the application-aware detection problem (10) and obtain near-optimal thresholds. Finally, we propose Algorithm 4 to solve the problem of application-aware detection in a time period. The algorithm implements a variation of simulated annealing algorithm and finds near-optimal detection thresholds.

### 5.1. Algorithm for Worst-Case Detection Error Loss Problem

We begin our analysis by studying the computational complexity of finding worst-case loss due to detection errors (9). To this end, we formulate the problem of finding a worst-case loss as a decision problem.

**Definition** **4** (Worst-Case Detection Error Problem (Decision Version)). *Given a set of sensors S, detection thresholds **τ**, residuals r, and desired loss $L^{*}$, determine whether there exists a detection error scenario that incurs the detection error loss of at least $L^{*}$.*

The following theorem establishes the computational complexity of finding a worst-case detection error.

**Theorem** **1.** *Worst-Case Detection Error Problem (WCDE) is NP-Hard.*

**Proof.**  We prove the above theorem using a reduction from a well-known NP-hard problem, the Maximum Independent Set Problem.

**Definition** **5** (Maximum Independent Set Problem (Decision Version)). *Given an undirected graph G=(V,E) and a threshold cardinality k, determine whether there exists an independent set of nodes (i.e., a set of nodes such that there is no edge between any two nodes in the set) of cardinality k.*

Given an instance of the Maximum Independent Set Problem (MIS), that is, a graph G=(V,E) and a threshold cardinality *k*, we construct an instance of the WCDE as follows:

- Let the set of sensors be S:=V.
- Let $p_{s}=0$ and $m_{s}=1$ for every sensor s∈S.
- For every sensor s∈S, let $\tau_{s}=\epsilon$ where ϵ<1, so that A=S and N=∅.
- Let $T\;P_{s}\left(\tau_{s}\right)=F\;P_{s}\left(\tau_{s}\right)=T\;N_{s}\left(\tau_{s}\right)=F\;N_{s}\left(\tau_{s}\right)=0.5$ for every sensor s∈S.
- Let the dimension of the control signal be |S|. For each element *i* of *u*, let $u_{i}\in \left\{0,1\right\}$.
- Let the utility function be $J\left(w,u\right)={∥w\circ u∥}_{1}$ if the non-zero elements in w form a non-empty independent set, and $-{∥u∥}_{1}$ otherwise.
- Finally, let the threshold loss be $L^{*}:={\left(\frac{1}{2}\right)}^{\left|S\right|}k$.

Clearly, the above reduction can be performed in polynomial time. Hence, it remains to show that the constructed instance of WCDE has a solution *if and only if* the given instance of MIS does.

*MIS then WCDE.* First, suppose that MIS has a solution, that is, there exists an independent set *I* of *k* nodes. We claim that the set fp=I and tp=S−I is a solution to WCDE. We have

$$\Delta J=J^{*}\left(p_{tp}\cup m_{fp}\right)-J\left(p_{tp}\cup m_{fp},U\left(p_{A}\right)\right))=J^{*}\left(m_{fp}\right)-J\left(m_{fp},<\;0\;{>}_{i\in 1}^{\left|S\right|}\right)={∥m_{fp}∥}_{1}-0=k$$

Since $\mathrm{Pr}\left(\tau ,tp,fp,tn,fn\right)={\left(\frac{1}{2}\right)}^{\left|S\right|}$ for any given sets of detection error, we obtain $L\left(\tau \right)={\left(\frac{1}{2}\right)}^{\left|S\right|}\cdot k$.

**Not MIS then Not WCDE.** Second, suppose that MIS has no solution, that is, every set of at least *k* nodes is non-independent. Then, we have that J(w,u)<k for every w; otherwise, there would exist a set of at least *k* nodes in *I* that are independent of each other, which would contradict our supposition. Then, since $\mathrm{Pr}\left(\tau ,tp,fp,tn,fn\right)={\left(\frac{1}{2}\right)}^{\left|S\right|}$, we conclude $L\left(\tau \right)<{\left(\frac{1}{2}\right)}^{\left|S\right|}\cdot k$. □

**Algorithm 2** Algorithm for Computing Worst-Case Loss.
1:**function**Worst_Loss(τ,m,p)2:    **for all** s∈S **do**3:        ($|m_{s}-p_{s}|\leq \tau_{s}$) ? N←N∪{s} : A←A∪{s}4:    **end for**5:    tp←A, fp←∅6:    tn←N, fn←∅7:    $L^{*}\leftarrow 0$8:    **while** tp≠∅ortn≠∅ **do**9:        $\left(C_{i},i\right)\leftarrow \max_{i\subseteq tp}C\left(\tau ,tp\backslash i,fp\cup \left\{i\right\},tn,fn\right)$10:        $\left(C_{j},j\right)\leftarrow \max_{j\subseteq tn}C\left(\tau ,tp,fp,tn\backslash j,fn\cup \left\{j\right\}\right)$11:        **if** $C_{i}<L_{j}$ **then**12:           $C\leftarrow C_{j}$13:           tn←tn\j14:           fn←fn∪{j}15:        **else**16:           $C\leftarrow C_{i}$17:           tp←tp\i18:           fp←fp∪{i}19:        **end if**20:        **if** $C^{*}<C$ **then**21:           $C^{*}\leftarrow C$22:        **else**23:           **return** $C^{*}$24:        **end if**25:    **end while**26:    **return** $C^{*}$27:
**end function**



We present Algorithm 2 which uses a greedy approach to obtain the worst-case loss due to detection errors. The algorithm starts considering a scenario of perfect detection, that is, tp=A, fp=∅, tn=N and fn=∅. In each iteration, the algorithm moves an element from either tp or tn to respectively fp or fn that maximally increases the utility loss. If no such element exists, the algorithm terminates with the best solution found so far.

The runtime of Algorithm 2 depends on the function *J*, which depends on the considered application. If there is an oracle that computes U(w) and J(w,U(w)) in constant time, the runtime of Algorithm 2 is linear with respect to |S|. That is, the runtime of Algorithm 2 is O(|S|).

### 5.2. Algorithm for Application Aware Anomaly Detection Problem

To solve the AAAD problem, we first prove the following lemma. The lemma shows that the problem is equal to the problem of selecting a set of normal sensors *N* and a set of anomalous sensors *A*, which has a much smaller search space than the original problem.

**Lemma** **1.** *For sensor s with residual $r_{s}$, the optimal threshold with respect to (10) satisfies $\tau_{s}\in \;\left\{0,r_{s},r_{s}^{+},M\right\}$.*

**Proof.**  We need to prove that for sensor *s* with residual $r_{s}$, the optimal threshold with respect to (10) is in the set $\left\{0,r_{s},r_{s}^{+},M\right\}$. First, let us recall that the optimal threshold is

$$\begin{array}{c} \tau^{*}\in \underset{\tau }{\mathrm{argmin}}\max_{\begin{array}{c} tp\subseteq A,tn\subseteq N\\ fp=A-tp\\ fn=N-tn \end{array}}\mathrm{Pr}\left(\tau ,tp,fp,tn,fn\right)\cdot \Delta J\;, \end{array}$$

where $\Delta J=J^{*}\left(p_{tp}\cup m_{fp}\cup m_{tn}\cup p_{fn}\right)-J\left(p_{tp}\cup m_{fp}\cup m_{tn}\cup p_{fn},U\left(p_{A}\cup m_{N}\right)\right)$.

Suppose there exists a set of optimal thresholds $\tau^{*}$ such that some of its elements are not in the set mentioned above. Let *s* be one such sensor, that is, $\tau_{s}^{*}\notin \left\{0,r_{s},r_{s}^{+},M\right\}$. First, let $0<\tau_{s}^{*}<r_{s}$. Clearly, $\Delta J\left(\tau^{*}\right)=\Delta J\left(\tau_{-s}\cup \left\{0\right\}\right)=\Delta J\left(\tau_{-s}\cup \left\{r_{s}\right\}\right)$. Then, we write $\frac{\mathrm{Pr}(\tau^{\prime },tp,fp,tn,fn)}{\mathrm{Pr}(\tau^{*},tp,fp,tn,fn)}=\frac{T\;P_{s}\left(\tau_{s}^{\prime }\right)}{T\;P_{s}\left(\tau_{s}^{*}\right)}\;>\;1$ if $\tau_{s}^{\prime }=0$, and so $\tau_{s}^{*}$ cannot be the optimal threshold if *s* is in the set of true positives. Also, $\frac{\mathrm{Pr}(\tau^{\prime },tp,fp,tn,fn)}{\mathrm{Pr}(\tau^{*},tp,fp,tn,fn)}=\frac{F\;P_{s}\left(\tau_{s}^{\prime }\right)}{F\;P_{s}\left(\tau_{s}^{*}\right)}>1$ if $\tau_{s}^{\prime }=r_{s}$, and so $\tau_{s}^{*}$ cannot be the optimal threshold if *s* is in the set of false positives either. Second, let $r_{s}^{+}<\tau_{s}^{*}<M$. Again, we have $\Delta J\left(\tau^{*}\right)=\Delta J\left(\tau_{-s}\cup \left\{r_{s}^{+}\right\}\right)=\Delta J\left(\tau_{-s}\cup \left\{M\right\}\right)$. Then, we write $\frac{\mathrm{Pr}(\tau^{\prime },tp,fp,tn,fn)}{\mathrm{Pr}(\tau^{*},tp,fp,tn,fn)}=\frac{T\;N_{s}\left(\tau_{s}^{\prime }\right)}{T\;N_{s}\left(\tau_{s}^{*}\right)}>1$ if $\tau_{s}^{\prime }=M$. Also, $\frac{\mathrm{Pr}(\tau^{\prime },tp,fp,tn,fn)}{\mathrm{Pr}(\tau^{*},tp,fp,tn,fn)}=\frac{F\;N_{s}\left(\tau_{s}^{\prime }\right)}{F\;N_{s}\left(\tau_{s}^{*}\right)}>1$ if $\tau_{s}^{\prime }=r_{s}^{+}$. This means that $\tau_{s}^{*}$ cannot be the optimal threshold if *s* is in the set of true negatives or false negatives either. This contradicts our supposition, and thus, $\tau_{s}\notin \left\{0,r_{s},r_{s}^{+},M\right\}$ can never be correct. This concludes our proof. □

**Algorithm 3** Algorithm for Design of Application-Aware Detector.
1:**function**Application_Aware(m,p)2:    N←S, A←∅3:    $L^{*}\leftarrow \infty$4:    **while** A≠S **do**5:        $\left(L,s\right)\leftarrow {\mathrm{argmin}}_{s\in N}Worst\_Loss\left(A\cup \left\{s\right\},N\backslash \left\{s\right\},m,p\right)$6:        **if** $L^{*}<L$ **then**7:           $L^{*}\leftarrow L$8:           A←A∪{s}9:           N←N\{s}10:        **else**11:           **return** $L^{*}$12:        **end if**13:    **end while**14:    **return** $L^{*}$15:
**end function**



Following the above lemma, we present Algorithm 3 to obtain application-aware detection thresholds. The algorithm begins by initializing all sensors as normal, that is, N=S and A=∅. In each iteration, the algorithm moves a sensor from *N* to *A*, which maximally decreases the worst-case loss. To compute the worst-case loss, Algorithm 2 is used.

Similar to the previous algorithm, the running time depends on the function *J* and the considered application. If there is an oracle that returns U(w) and J(w,U(w)) in constant time, the runtime of Algorithm 3 is $O(|S{|}^{2})$.

### 5.3. Algorithm for Application-Aware Anomaly Detection in a Time Period

We present Algorithm 4 which solves the problem of application-aware detection in a time period *T* (2). The algorithm is based on a variation of simulated annealing algorithm, and finds near-optimal thresholds τ. The idea is to start with an arbitrary solution τ and improving it iteratively. In each iteration, we generate a new candidate solution $\tau^{\prime }$ in the neighborhood of τ. If the candidate solution $\tau^{\prime }$ is better in minimizing the loss, then the current solution is replaced with the new one. However, if $\tau^{\prime }$ increases the loss, the new solution replaces the current solution with only a small probability. This probability depends on the difference between the two solutions in terms of loss as well as a temperature parameter which is a decreasing function of the number of iterations. These random replacements decreases the likelihood of getting stuck in a local minimum.

**Algorithm 4** Algorithm for Design of the Application-Aware in a Time Period.
1:**Input:**m, p2:**Initialize:**τ, n←1, $T_{0}$3:
L(τ)←Worst_Loss(τ,m,p)
4:
**while** $n\leq n_{\max}$ **do**
5:    $\tau^{\prime }\leftarrow Perturb\left(\tau ,n\right)$6:    $L\left(\tau^{\prime }\right)\leftarrow Worst\_Loss\left(\tau^{\prime },m,p\right)$7:    $c\leftarrow e^{(L\left(\tau^{\prime }\right)-L\left(\tau \right))/T}$8:    **if** $\left(L\left(\tau^{\prime }\right)<L\left(\tau \right)\right)\;\vee \;\left(\mathrm{rand}\left(0,1\right)\leq c\right)$ **then**9:        $\tau \leftarrow \tau^{\prime }$, $L\left(\tau \right)\leftarrow L\left(\tau^{\prime }\right)$10:    **end if**11:    $T\leftarrow T_{0}\cdot e^{-\beta n}$12:    n←n+113:
**end while**
14:
**return** τ



In Algorithm 4, Perturb(τ,n) defines the neighborhood of τ in the *n*th iteration, from which $\tau^{\prime }$ is randomly sampled. More specifically, Perturb(τ,n) means that each $\tau_{s}$ in τ is replaced by $\tau_{s}^{\prime }=\tau_{s}+\Delta \tau_{s}$. Here, for each s∈S, $\Delta \tau_{s}$ is randomly picked from the uniform distribution over $\left[-\alpha \left(\frac{n_{\max}-n}{n_{\max}}\right)\;,\;\alpha \left(\frac{n_{\max}-n}{n_{\max}}\right)\right]$ for some $\alpha \in R_{+}$. Moreover, since $\tau_{s}^{\prime }$ is nonnegative, we replace it with 0 if $\tau_{s}^{\prime }<0$.

## 6. Special Cases

In this section, we consider two special cases for the AAAD problem (10). The first special case is single detector, which means that either there is a single detector in the system or each detector is optimized independently of other detectors. The second special case is detectors with equal thresholds, where there are multiple detectors that have the same thresholds.

### 6.1. Single Detector

Consider a scenario where |S|=1. This means that either there is a single detector in the system, or each detector is optimized independently and irrespective of other detectors. Let S={a} be the considered sensor, and let $r_{a}=\left|p_{a}-m_{a}\right|$ be the residual of the sensor at a timestep. First, we consider a threshold $\tau_{a}^{\prime }$ where $r_{a}>\tau_{a}^{\prime }$ for this sensor. This threshold results in a detection alert, and so the set of marked-anomalous sensors becomes A={a} and the set of marked-normal sensors becomes N=∅. Next, to find the worst-case detection error loss (9) for this threshold, there are two possibilities for tp: (1) tp={a} and fp=∅, and (2) tp=∅ and fp={a}. For tp={a}, i.e., no detection error, we can write

$$C\left(\tau_{a}^{\prime },\left\{a\right\},\emptyset ,\emptyset ,\emptyset \right)=T\;P\left(\tau_{a}^{\prime }\right)\cdot \;\left(J\left(p_{a},U\left(p_{a}\right)\right)-J\left(p_{a},U\left(p_{a}\right)\right)\right)=0\;.$$

For the second scenario, i.e., tp=∅ and fp={a}, we should have used the measurement $m_{a}$ but we used the prediction $p_{a}$, and so the expected utility loss is

$$C\left(\tau_{a}^{\prime },\emptyset ,\left\{a\right\},\emptyset ,\emptyset \right)=F\;P\left(\tau_{a}^{\prime }\right)\cdot \;\left(J\left(m_{a},U\left(m_{a}\right)\right)-J\left(m_{a},U\left(p_{a}\right)\right)\right)\;.$$

Therefore, the worst-case utility loss for the threshold $\tau_{a}^{\prime }$, where $r_{a}>\tau_{a}^{\prime }$, is obtained using

$$L\left(\tau_{a}^{\prime }\right)=\max C\left(\tau_{a}^{\prime },\emptyset ,\left\{a\right\},\emptyset ,\emptyset \right)=C\left(r_{a}^{-},\emptyset ,\left\{a\right\},\emptyset ,\emptyset \right)$$

Next, we consider a threshold $\tau_{a}^{\prime \prime }$ such that $r_{a}\leq \tau_{a}^{\prime \prime }$. This threshold results in no detection alert, and so A=∅ and N={a}. To compute the worst-case detection error loss, there are two possibilities for detection error: (1) tn={a} and fn=∅, and (2) tn=∅ and fn={a}. Similar to the above scenario, for the first case which corresponds to no detection error, we obtain ΔJ=0 and so $C(\tau_{a}^{\prime \prime },\emptyset ,\emptyset ,\left\{a\right\},\emptyset )=0$. For the second case, we obtain

$$C\left(\tau_{a}^{\prime \prime },\emptyset ,\emptyset ,\emptyset ,\left\{a\right\}\right)=F\;N\left(\tau_{a}^{\prime \prime }\right)\cdot \;\left(J\left(p_{a},U\left(p_{a}\right)\right)-J\left(p_{a},U\left(m_{a}\right)\right)\right)\;.$$

Therefore, the worst-case detection error loss for the threshold $\tau_{a}^{\prime \prime }$, where $r_{a}\leq \tau_{a}^{\prime \prime }$, is

$$L\left(\tau_{a}^{\prime \prime }\right)=\max C\left(\tau_{a}^{\prime \prime },\emptyset ,\emptyset ,\emptyset ,\left\{a\right\}\right)=C\left(r_{a},\emptyset ,\emptyset ,\emptyset ,\left\{a\right\}\right)\;.$$

The application-aware detector selects the threshold $\tau_{a}^{*}\in \left\{r_{a}^{-},r_{a}\right\}$ that solves

$$\min(\;C\left(r_{a}^{-},\emptyset ,\left\{a\right\},\emptyset ,\emptyset \right),C\left(r_{a},\emptyset ,\emptyset ,\emptyset ,\left\{a\right\}\right)\;)\;.$$

In other words, the optimal threshold is

(13) $$\begin{array}{c} \tau_{a}=\left\{\begin{array}{cc} r_{a}^{-} & \;\mathrm{if}\;C\left(r_{a}^{-},\emptyset ,\left\{a\right\},\emptyset ,\emptyset \right)\leq C\left(r_{a},\emptyset ,\emptyset ,\emptyset ,\left\{a\right\}\right)\\ r_{a} & \;\mathrm{otherwise} \end{array}\right.. \end{array}$$

### 6.2. Detectors with Equal Thresholds

We consider a case where all detectors have equal thresholds. Let $\bar{\tau }$ represent this threshold value, that is, $\bar{\tau }=\tau_{1}=\ldots =\tau_{d}$. Next, let $r_{1},r_{2},\ldots ,r_{d}$ be the residual values. The result below is a direct consequence of Lemma 1.

**Corollary** **1.** *The optimal threshold $\bar{\tau }$ for detectors with equal thresholds belongs to the following set*

$$\mathrm{SolutionSpace}=\{0,r_{1}^{-},r_{1}^{+},r_{2}^{-},r_{2}^{+}\ldots ,r_{d}^{-},r_{d}^{+},M\}\;.$$

Based on the above corollary, since the solution space is finite, we can find the optimal thresholds by a linear-time search, as presented by Algorithm 5.

**Algorithm 5** Application-Aware Detector for Equal Thresholds.
**Input:**m, p
1:
r←|m−p|
2:
**for all** $\bar{\tau }\in \mathrm{SolutionSpace}$ **do**
3:    $(L\left(\bar{\tau }\right),\bar{\tau })\leftarrow$Worst_Loss($\bar{\tau },m,p$)4:
**end for**
5:
${\bar{\tau }}^{*}\leftarrow \mathrm{argmin}\left(L\left(\bar{\tau }\right),\bar{\tau }\right)$
6:
**return** ${\bar{\tau }}^{*}$



## 7. Evaluation

In the preceding sections, we presented general computational results and general-purpose algorithms for the AAAD problem. In this section, to demonstrate the practical application of our AAAD framework, we provide numerical results on our running example. In particular, we apply our approach to a case study of max-pressure control of traffic signals in a traffic network. First, we construct regression-based anomaly detectors for traffic sensors, and we generate the trade-off curves for their performance. Then, we implement the AAAD approach, and evaluate its performance compared to a baseline “application-unaware” detector configuration. Throughout this section, we use SUMO (Simulation of Urban MObility), which is a micro simulator for traffic applications [27].

### 7.1. Traffic Network

**Figure 4:**
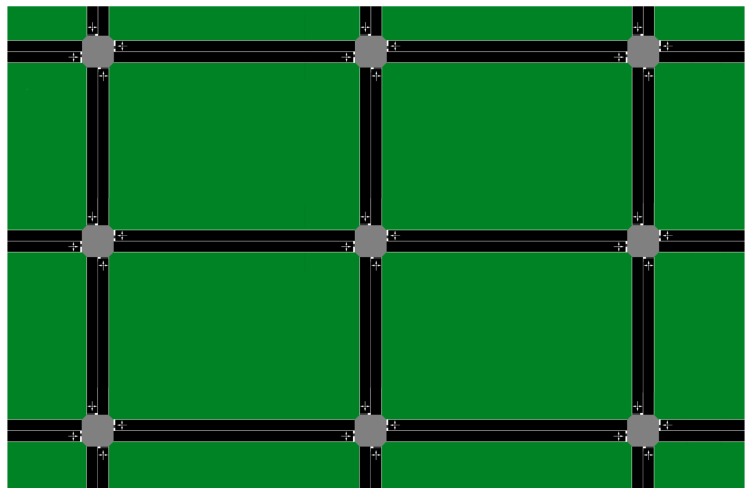
The 3-by-3 grid.

**Figure 5:**
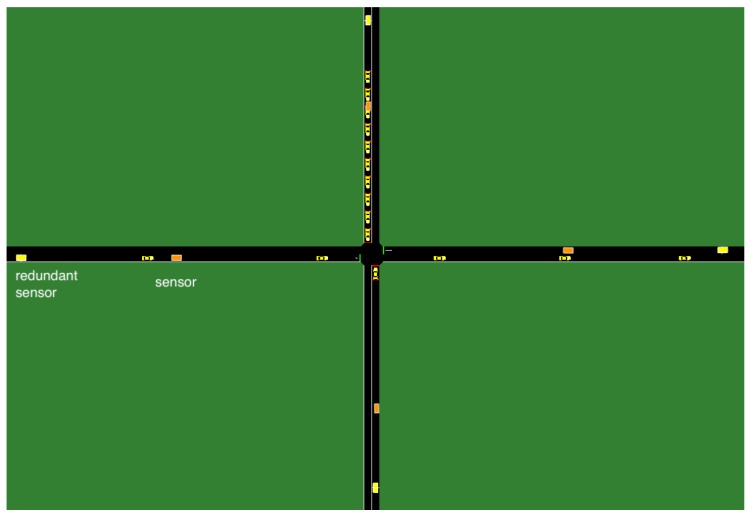
Each intersection in the 3-by-3 grid. For illustration, a critical and a redundant sensor are shown in the figure. For each lane, a second redundant sensor is placed with twice as much distance as the distance between the first redundant sensor and the critical sensor. All 8 total redundant sensors are used to predict the value of a sensor.

Consider a traffic network in a 3-by-3 grid with a total of 9 intersections, as show in Figure 4. We perform our numerical evaluation in a simulated environment, where we have accurate ground truth regarding sensor faults. Each intersection connects 4 standard two-way lanes with four possible movements {EW, WE, NS, SN}, as shown in Figure 5. Traffic volume of each movement is monitored by the set of sensors $S=\{s_{EW},s_{WE},s_{NS},s_{SN}\}$. The sensors send traffic measurements $m\;=\;\{m_{EW},m_{WE},m_{NS},m_{SN}\}$ at each timestep. Each traffic signal has two phases $\Phi =\{\phi_{\left\{EW,WE\right\}},\phi_{\left\{NS,SN\right\}}\}$. The max-pressure controller computes the optimal stage $u^{*}\in \Phi$ using (2).

The utility (i.e., pressure-release) function of the traffic network can be written as sum of the pressure-release of each individual intersection, and for each intersection, the utility depends only on its corresponding lanes. This means that maximizing the pressure-release of the traffic network is equal to maximizing the pressure-release of individual intersections, and so the application-aware threshold of each intersection can be designed independently of other intersections. Based on this observation, in what follows, we discuss how the detector is designed for an intersection and then extend it to all intersections.

#### 7.1.1. Anomaly Model

Traffic measurements may be anomalous due to failures or other undesired events. To simulate the negative effects of anomalies on the system, we consider several realistic anomaly models [28],

1. *Overcount*: Additive error equal to 3% to 7% of the actual values, i.e., $e_{s}\left(k\right)=u_{s}a_{s}\left(k\right)$ and $0.03\leq u_{s}\leq 0.07$.
2. *Undercount*: Subtractive error equal to 7% to 13% of the actual values, i.e., $e_{s}\left(k\right)=u_{s}a_{s}\left(k\right)$ and $-0.13\;\leq \;u_{s}\;\leq \;-0.07$.
3. *Gaussian Noise*: Error with zero mean and standard deviation σ=15 to σ=35, i.e., $e_{s}\left(k\right)\;∼N\left(0,\sigma^{2}\right)$ and 15≤σ≤35.

### 7.2. Regression-Based Detector and Trade-Off Curves

To protect the system against anomalies, we construct anomaly detectors. We suppose there are 8 (2 on each side) redundant sensors that are adjacent to the four critical sensors mentioned above. We use the values of these sensors to design regression-based anomaly detectors for the critical sensors. As discussed in the preceding sections, such detector consists of two main components: (1) Predictor and (2) Statistical Test.

#### 7.2.1. Predictor

We collect simulation data that represents the traffic behavior under normal operation. We simulate the network for 4 h considering a Poisson distribution as the demand for each movement. We collect sensor measurements in 10-s aggregates. The data from the first 2 h is used to train the predictors, and the data from the remaining 2 h is used to obtain the trade-off curves. Following our previous discussion, for each predictor, we use the current value of the 8 redundant sensors as the features. Then, we train the predictor using linear regression algorithm. We obtain the performance metrics $MSE_{train}=2.14$ and $MSE_{test}=2.87$. Please note that more complex regression algorithms (e.g., Gaussian Processes [24]) can be used as well; however, we obtained satisfactory result with a simple linear regression model.

#### 7.2.2. Trade-off Curve

**Figure 6:**
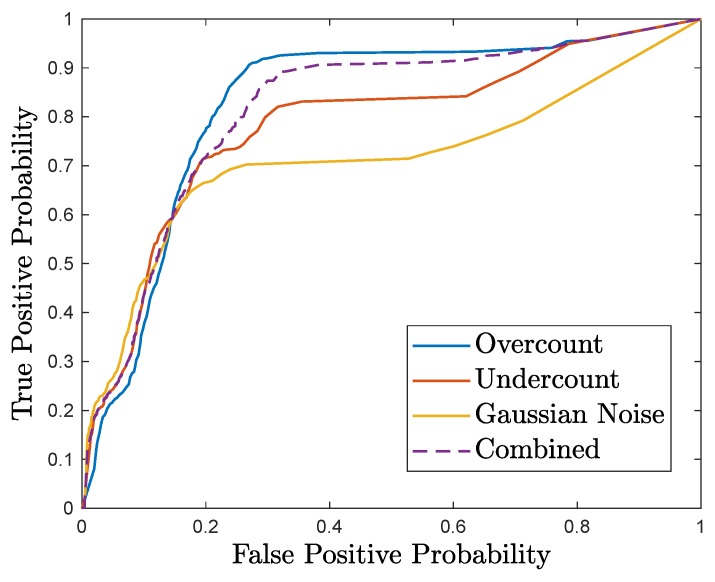
Trade-off between true positive and false positive errors for $s_{EW}$.

To generate the trade-off curve, first, we simulate the anomalies on the test data, and evaluate the performance of the detector by counting the number of true positives and false negatives. Similarly, we simulate the system under normal operation and evaluate the performance of the detector to obtain the number of true negatives and false positives. We repeat the steps while varying the detection threshold in order to obtain the trade-off curve (i.e., true positive probability as a function of false positive probability). Figure 6 shows the resulting trade-off curve.

### 7.3. Application-Aware Anomaly Detector

Given the trade-off curve, we implement the application-aware anomaly detector by finding the detection thresholds that minimize the worst-case expected utility loss. First, we show how the optimal threshold can be computed at each single timestep. Then, we present the results.

#### 7.3.1. Computing the Optimal Threshold

To show how the optimal threshold is computed at each timestep, suppose $m_{NS}=p_{NS}=15$ at a given timestep. Further, suppose there is no traffic on SN and WE. If $w_{EW}\geq 15$, the max-pressure controller selects the stage $u=\phi_{\left\{EW,WE\right\}}$, and otherwise, it selects $u=\phi_{\left\{NS,SN\right\}}$. Next, consider the following scenarios for $m_{EW}$ and $p_{EW}$:

1. $m_{EW}=30$ and $p_{EW}=10$. In this case, the threshold that solves the following equation is the optimal threshold: $\min\left(C\left(20^{-},\emptyset ,\left\{s_{EW}\right\},\emptyset ,\emptyset \right),C\left(20,\emptyset ,\emptyset ,\emptyset ,\left\{s_{EW}\right\}\right)\right)$, where $C\left(20^{-},\emptyset ,\left\{s_{EW}\right\},\emptyset ,\emptyset \right)=F\;P\left(20^{-}\right)\cdot \;\left(J^{*}\left(30\right)-J\left(30,U\left(10\right)\right)\right)=30-15=15\;,$ and $C\left(20,\emptyset ,\emptyset ,\emptyset ,\left\{s_{EW}\right\}\right)=F\;N\left(20\right)\cdot \;\left(J^{*}\left(10\right))-J\left(10,U\left(30\right)\right)\right)=15-10=5\;.$ Thus, the optimal threshold is $\tau^{*}=20$. To see why this is the optimal threshold, note that if τ=20, then there will be no detection alert and the measurement ($m_{EW}=30$) will be used in computing the optimal control input (which is EW). If this is incorrect (due to a false negative), then $p_{SE}=10$ is the actual value. In this case, the pressure-release of $\phi_{\left\{NS,SN\right\}}$ will be 10. In the perfect detection case (no detection error), we could have obtained 15 by selecting NS. Thus, the detection error loss is 15−10=5. On the other hand, if $\tau =20^{-}$, the prediction will be used by the controller. This results in the movement NS being selected. However, if there is a false positive, which means $m_{EW}=30$ is the actual value, then EW should have been selected as the optimal stage, which could have obtained a utility of 30. Thus, the utility loss in this case is 30−15=15, which is larger than the utility loss for the other threshold.
2. $m_{EW}=30$, $p_{EW}=10$, and $a_{NS}=25$. The application-aware detector computes the minimum of $F\;P^{-}\left(20\right)\cdot \;\left(J^{*}\left(30\right)-J\left(30,U\left(10\right)\right)\right)=30-25=5$, and $F\;N\left(20\right)\cdot \;\left(J^{*}\left(10\right)-J\left(10,U\left(30\right)\right)\right)=25-10=15$. In this case, the threshold $\tau =20^{-}$ is the optimal threshold.
3. $m_{EW}=30$, $p_{EW}=25$, and $a_{NS}=15$. The application-aware detector computes $F\;P\left(20^{-}\right)\cdot \;\left(J^{*}\left(30\right)-J\left(30,U\left(25\right)\right)\right)=30-30=0$ and $F\;N\left(20\right)\cdot \;\left(J^{*}\left(25\right)-J\left(25,U\left(30\right)\right)\right)=25-25=0$, which means that both thresholds are optimal. The same results can be obtained if $a_{NS}\geq m_{EW}$ and $a_{NS}\geq p_{EW}$.

#### 7.3.2. Comparison

We now compare our application-aware detector configuration to a baseline detector configuration, which does not take into account the underlying CPS application. For a fair comparison, the threshold of the baseline detector is selected such that it attains the same false-alarm probability as the application-aware anomaly detector. That is, we first calculate the total number of false alarms for the application-aware detector configuration, and then select the threshold that obtains the same false-alarm probability for the baseline configuration. In our numerical example, in 2 h of evaluation time during which each of the anomalies may occur with probability of 0.05, each application-aware detector had on average a false-alarm probability of 0.047. The threshold values for the application-aware detector varied from 1.3 to 26.5 with a mean of 5.6. The threshold for the baseline detector was selected to be 3.9.

**Figure 7:**
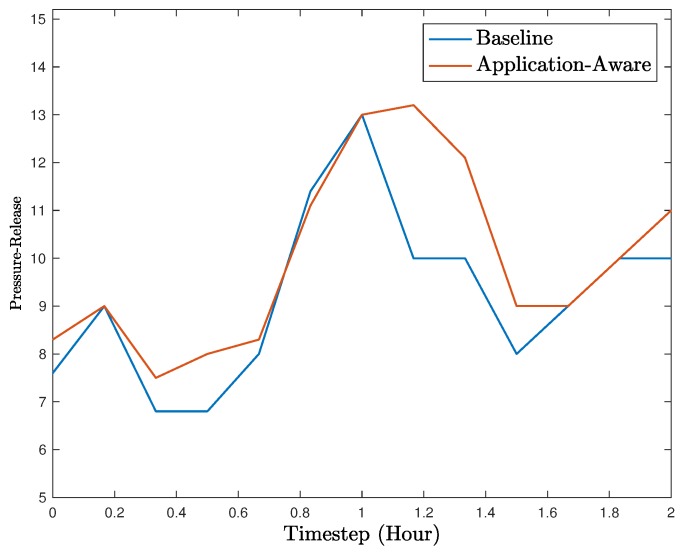
Utility (i.e., pressure-release) during a 2-h interval.

Figure 7 shows the pressure-release comparison between the two cases during the 2-h interval. Each tick in the figure aggregates the results from 12 min (i.e., 72 timesteps). Based on the results, the application-aware detector performs better than the baseline detector in most cases. The baseline detector performs only slight better in the aggregated timestep at 0.8, which could be due to detection errors by the application-aware detector. However, in the rest of the 2-h period, the proposed detector perform significantly better.

## 8. Conclusions

We presented the application-aware anomaly detection framework for detecting anomalies in sensor measurements in cyber-physical systems. An application-aware anomaly detector configures itself such that the application performance in the presence of detection errors is as close as possible to the performance that could have been obtained if there were no detection errors. We formulated and studied the problem of optimal, application-aware configuration of an existing detector. We evaluated our result using a case study of real-time control of traffic signals, and showed that our application-aware detector configuration significantly outperforms the baseline.

## Figures and Tables

**Table 1:** List of Symbols.

Symbol	Description
*S*	Set of sensors
$a_{s}$	Actual value for sensor *s*
$m_{s}$	Measured value for sensor *s*
$p_{s}$	Predicted value for sensor *s*
$T\;P_{s}\left(\tau \right)$	True positive probability of the detector for sensor *s* given detection threshold τ
$F\;P_{s}\left(\tau \right)$	False positive probability of the detector for sensor *s* given detection threshold τ
$T\;N_{s}\left(\tau \right)$	True negative probability of the detector for sensor *s* given detection threshold τ
$F\;N_{s}\left(\tau \right)$	False negative probability of the detector for sensor *s* given detection threshold τ
$w_{s}$	Recovered measurement transmitted to the controller for sensor *s*
$r_{s}$	Residual signal for sensor *s*
